# Biomineralization in Cave Bacteria—Popcorn and Soda Straw Crystal Formations, Morphologies, and Potential Metabolic Pathways

**DOI:** 10.3389/fmicb.2022.933388

**Published:** 2022-07-01

**Authors:** Keegan Koning, Richenda McFarlane, Jessica T. Gosse, Sara Lawrence, Lynnea Carr, Derrick Horne, Nancy Van Wagoner, Christopher N. Boddy, Naowarat Cheeptham

**Affiliations:** ^1^Department of Biology, Faculty of Science, Thompson Rivers University, Kamloops, BC, Canada; ^2^Department of Chemistry and Biomolecular Sciences, University of Ottawa, Ottawa, ON, Canada; ^3^The University of British Columbia Bioimaging Facility, Biological Sciences Building, Vancouver, BC, Canada; ^4^Department of Physical Sciences, Faculty of Science, Thompson Rivers University, Kamloops, BC, Canada

**Keywords:** cave microorganisms, cave microbiology, geomicrobiology, MICP, urease, biomineralization, speleothems, cave formation

## Abstract

Caves are extreme, often oligotrophic, environments that house diverse groups of microorganisms. Many of these microbes can perform microbiologically induced carbonate precipitation (MICP) to form crystalline secondary cave deposits known as speleothems. The urease family is a group of enzymes involved in MICP that catalyze the breakdown of urea, which is a source of energy, into ammonia and carbonate. Carbonate anions are effluxed to the extracellular surface of the bacterium where it then binds to environmental calcium to form calcium carbonate which then continues to grow in crystal form. Here, we studied bacterial communities from speleothems collected from the Iron Curtain Cave (ICC) in Chilliwack, B.C., Canada, to characterize these organisms and determine whether urease-positive (U+) bacteria were present in the cave and their potential impact on speleothem formation. The ICC is a carbonate cave located on the northside of Chipmunk Ridge, presenting a unique environment with high iron content sediment and limestone structures throughout. With six pools of water throughout the cave, the environment is highly humid, with temperatures ranging between 4 and 12°C depending on the time of year. Ninety-nine bacterial strains were isolated from popcorn (PCS) and soda straw (SSS) speleothems. These isolates were screened for urease enzymatic activity, with 11 candidates found to be urease-positive. After incubation, species-specific crystal morphologies were observed. Popcorn speleothem provided more bacterial diversity overall when compared to soda straw speleothem when examined under a culture-based method. Nearly twice as many U+ isolates were isolated from popcorn speleothems compared to soda straw speleothems. The U+ candidates were identified to the genus level by 16S rRNA analysis, and two isolates underwent whole-genome sequencing. Two novel species were identified as *Sphingobacterium* sp. PCS056 and *Pseudarthrobacter* sp. SSS035. Both isolates demonstrated the most crystal production as well as the most morphologically dissimilar crystal shapes in broth culture and were found to produce crystals as previously observed in both agar and broth media. The results from this study are consistent with the involvement of urease-positive bacteria isolated from the ICC in the formation of cave speleothems. 16S rRNA sequencing revealed a diverse set of microbes inhabiting the speleothems that have urease activity. Whole-genome sequencing of the two chosen isolates confirmed the presence of urease pathways, while revealing differences in urease pathway structure and number. This research contributes to understanding microbial-associated cave formation and degradation, with applications to cave conservation, microbiota composition, and their role in shaping the cave environment.

## Introduction

Caves are generally defined as areas of subterranean space which occur naturally in the environment and which are accessible by living organisms. Caves can be characterized by many variables; however, typically, they are classified by the type of rock and the formation process of the caves (Northup and Lavoie, 2001). Limestone caves are formed via acid dissolution, when groundwater flows through the soil and rock (vadose) layers, where it interacts with carbon dioxide (CO_2_) gas and produces carbonic acid (H_2_CO_3_), which slowly dissolves the carbonate rock. In sulfuric acid dissolution, hydrogen sulfide (H_2_S) gas rises from rock fissures (narrow openings of rocks, commonly found in tectonically active areas) and reacts with atmospheric oxygen to form sulfuric acid (H_2_SO_4_) that dissolves the carbonate rock to form caves (Palmer, 1991; Northup and Lavoie, 2001). Furthermore, decomposition of organic matter by microorganisms can be a source of sulfuric acid dissolution (Beolchini et al., 2017).

Of particular interest are limestone caves, which can be found throughout British Columbia, Canada (Pike et al., 2010). The Iron Curtain Cave (ICC) is a restricted-access cave located in the Chilliwack River Valley (CRV), British Columbia, Canada. The cave is hosted by the Chilliwack Group which includes Pennsylvanian Permian basic volcanic rocks, Jurassic pelite, sandstone, and limestone karst (Saunders et al., 1987). The ICC is decorated by several speleothems, including popcorn, soda straw, stalactites, stalagmites, and bacon strips, with a large curtain of iron-containing minerals which gave the cave its name. Cave temperature ranges between 4 and 12°C annually (Ghosh et al., 2017) with relative humidity at greater than 90% (Carlyle-Moses, personal correspondence 2018).

Though chemical mechanisms of cave formation and cave architecture have been well researched, more recent studies have been focused on the biological mechanisms of cave formation. Engel et al. (2004) demonstrated that microbial mats, containing species of the class Epsilonproteobacteria, could oxidize hydrogen sulfide (H_2_S) into H_2_SO_4_, thus increasing the dissolution of limestone, while Barton et al. (2007) demonstrated that bacteria of the genus *Massilia* from a New Mexico cave system could utilize aqueous carbohydrates originating from surface water, to produce acids as a metabolic byproduct, dissolving carbonate bedrock in the caves.

Speleothems are distinct secondary cave mineral deposits/structures (Northup and Lavoie, 2001; Mason et al., 2016) that take many macroscopic and microscopic forms and are often composed of calcium carbonate polymorphs (Palmer, 1991; Baskar et al., 2014; Dhami et al., 2018). Speleothems are known to form by two mechanisms: abiotic (chemical) formation where water saturated with calcium interacts with dissolved organic carbon forming calcareous crystal structures (Dreybrodt, 1999; Banks et al., 2010; Szymczak and Ladd, 2011) and/or biotic formation by microorganisms that actively extrude carbonate extracellularly which then interacts with calcium saturated water to form calcareous crystal structures (Northup and Lavoie, 2001; Banks et al., 2010). As of 2001, there are 38 official speleothem types recognized by specialists at the forefront of speleology (Northup and Lavoie, 2001). Soda straw speleothems (SSS) precipitate from drip water points with water flowing down the center of the straw. At the tip of the straw, carbon dioxide (CO_2_) degasses which can allow calcium carbonate precipitation (Paul et al., 2013). Popcorn speleothem (PCS) form on cave walls, stalagmites, and stalactites by the evaporation of water and when airflow is present (Warren et al., 1990). In this study, PCS and SSS speleothems were sampled to study their bacterial profile and its ability to biologically form crystal structures that contribute to cave formation and degradation. From this, 11 such isolates demonstrated various degrees of crystal production, with varying morphologies.

Microbially induced carbonate precipitation (MICP) is a form of biomineralization and is a natural process of carbonate precipitation, occurring worldwide and mediated primarily by bacteria. MICP occurs through three primary pathways: biologically induced mineralization (BIM), biologically mediated mineralization (BMM), or biologically controlled mineralization (BCM) (Castanier et al., 1999; Banks et al., 2010; Castro-Alonso et al., 2019; Seifan and Berenjian, 2019). Caves are often defined as oligotrophic and highly mineralized environments and can be further characterized as being devoid or nearly devoid of natural light (Cheeptham et al., 2013; Seifan and Berenjian, 2019). Due to these environmental pressures, bacteria utilize alternative pathways for energy production, and the ability for MICP to occur depends on four variables: (1) The pH of the environment, (2) the abundance of nucleation sites, (3) the concentration of calcium ions, and (4) dissolved inorganic carbon (Chou et al., 2011; Seifan and Berenjian, 2019).

MICP is a well-documented metabolic process which contributes to the formation of secondary cave deposits known as speleothems (Castanier et al., 1999; Stocks-Fischer et al., 1999; Northup and Lavoie, 2001; Engel et al., 2004; Banks et al., 2010; Cheeptham et al., 2013). One common metabolic pathway that leads to MICP is the ureolysis pathway, where bacteria catalyze urea found in the environment, mediated by urease enzymes, producing carbonate as a byproduct. In this instance, the carbonate is secreted extracellularly, where it interacts with calcium ions that have adsorbed to the cell by the following chemical pathways (Northup and Lavoie, 2001; Ferris et al., 2003; Banks et al., 2010; Chou et al., 2011; Castro-Alonso et al., 2019):


$$C\,O\,{\left(N\,H_{2}\right)}_{2}+H_{2}\to N\,H_{2}\,C\,O\,O\,H+N\,H_{3}$$

$$N\,H_{2}\,C\,O\,O\,H+H_{2}\,O\to N\,H_{3}+H_{2}\,C\,O_{3}$$

$$H_{2}\,C\,O_{3}↔H\,C\,O_{3}^{-}+H^{+}$$

$$2\,N\,H_{3}+2\,H_{2}\,O\to 2\,N\,H_{4}^{+}+2\,O\,H$$

$$H\,C\,O_{3}^{-}+H^{+}+2\,N\,H_{4}^{+}+2\,O\,H\to {\left(C\,O_{3}\right)}^{2-}+2\,N\,H_{4}^{+}+2\,H_{2}\,O$$

$$C\,a^{2+}+C\,e\,l\,l\,S\,u\,r\,f\,a\,c\,e\to C\,e\,l\,l\,S\,u\,r\,f\,a\,c\,e-C\,a^{2+}$$

$$C\,e\,l\,l\,S\,u\,r\,f\,a\,c\,e-C\,a^{2+}+{\left(C\,O_{3}\right)}^{2-}\to C\,e\,l\,l-C\,a\,C\,O_{3}$$

When carbonate and calcium concentrations are at their saturation points, calcium carbonate crystals can form. The two most thermodynamically stable crystal structures common to MICP are aragonite and calcite (Stocks-Fischer et al., 1999; Hammes and Verstraete, 2002; Banks et al., 2010; Seifan and Berenjian, 2019) with vaterite acting as a metastable phase transient intermediate which favors the more stable aragonite and calcite (Christy, 2017). Bacteria that perform MICP then become self-fossilized and act as nucleation sites for further calcium carbonate crystal growth, which in turn aids in the process of speleothem formation (Palmer, 1991; Northup and Lavoie, 2001; Türkgenci and Dogruöz Güngör, 2011; Seifan and Berenjian, 2019; Lyu et al., 2021).

Urease enzymes are broadly distributed throughout the bacterial domain, always occurring as multimeric proteins with nickel incorporated into the enzyme structure (Mobley et al., 1995; Konieczna et al., 2013). Most bacteria have three different polypeptides making up the urease enzyme, with different protein structures present in different organisms (Konieczna et al., 2013). In many bacterial species, the alpha, beta, and gamma subunits encoded by genes *ureA, ureB*, and *ureC* form a trimer of trimers (Benini et al., 1999). In addition, three accessory proteins, UreD, UreF, and UreG, are urease molecular chaperones that are dependent on GTP binding and hydrolysis by UreG (Soriano and Hausinger, 1999), while a fourth accessory protein, UreE, coordinates nickel in the active site of the enzyme (Colpas and Hausinger, 2000; Soriano et al., 2000; Mulrooney et al., 2005).

This study aimed to determine whether cave bacteria isolated from popcorn and soda straw speleothems were involved in MICP and to simulate crystal production and speleothem formation in the lab setting. This evidence demonstrates that these isolated cave bacteria are mainly involved in and contribute to the growth of cave speleothems. In addition, based on analysis of the genome to determine urease enzyme copies present, we sought to connect the ureolytic pathway to microbially induced carbonate precipitation for these cave bacteria and determine which strains have a more prominent role in shaping the cave environment.

## Materials and Methods

### Sample Collection

Samples were collected from two distinct areas of the ICC as shown in Supplementary Figure 1. PCS and SSS samples were collected into 50 mL conical tubes. PCS samples were scraped into the tubes using a sterilized scoopula, while SSS was collected by gently breaking off a speleothem into the tube with sterilized forceps. Temperature readings were taken at each sample site ranging from 8.8 to 9.4°C. Examples of the speleothems collected within the cave can be found in Supplementary Figure 2.

### Bacterial Isolation

Fresh PCS and SSS samples were weighed to a wet weight of 1 g. Each speleothem was then transferred to a sterilized mortar and pestle and ground to a paste. The paste was transformed into a slurry by adding 2 mL of distilled H_2_O. The slurry was transferred to a 2.5-mL microfuge tube. Microfuge tubes were closed and hand-shaken for 2 min. Microfuge tubes were then placed in a rotating incubator at 9°C set to 100 revolutions per minute (RPM) for 1 h. Samples were removed from the rotating incubator and left to stand for 5 min. A series of serial dilutions (log_10_) were performed with sequential dilutions ranging from 10^0^ through to 10^–4^. For each dilution, 100 μL of the sample was pipetted and spread onto 22-mL agar plates (in triplicate) of the following media types: nutrient agar (NA), ten times diluted nutrient agar (d_10_NA), and Reasoner’s 2-A agar (R2A). Plates were then left to dry before incubation at 9°C until distinguishable colonies had formed.

### Bioprecipitation and Urease Activity

B4 precipitation medium (Boquet et al., 1973) was used with specific modifications which included substituting the source of calcium from the original calcium acetate [Ca(C_2_H_3_O_2_)_2_] to one of the following: calcium nitrate (CaNO_3_), calcium citrate [Ca_3_(C_6_H_5_O_7_)_2_], and calcium chloride (CaCl_2_) all at 0.25% w/v. The modification of the calcium salt component of the B4 medium aimed to test calcium water solubility and the availability of aqueous calcium ions to interact with precipitated carbonate on the extracellular surface of the bacterial isolates, that is, to determine whether one calcium salt increased the rate of biocrystallization, over another. Each isolated bacterium was then inoculated onto the modified B4 agar medium in 33 mm thick agar plates, to prevent premature drying due to lengthy incubation period. A container of diH_2_O water was placed in the incubator to increase humidity. Modified B4 plates were inoculated, streaking one isolated colony from each of the pure culture plates (NA, R2A, d_10_NA). Plates were inverted and incubated at 9°C until crystals formed.

To determine urease activity, solid agar slants were prepared and poured following the manufacturer’s instructions [BBLTM Urea Agar Base Concentrate 10× (BD, Franklin Lakes, New Jersey, United States]. Urea base concentrate was filtered through a 0.22 μm polyethersulfone (PES) filter to a final volume of 5.0 mL using an Omnispense pump (Thermo Fisher, Waltham, United States). Inoculated slants were incubated at 9°C until bacterial growth was observed. Isolates demonstrating urease activity were then inoculated onto 114 mm plates containing modified B4 media. Plates were inverted and incubated at 9°C. Daily observation occurred for the first 30 days and then weekly for a further 9 weeks until crystals were detected.

To determine crystal formation in an aqueous environment, 40.0 mL of MICP broth [0.25% Ca(C_2_H_3_O_2_)_2_, 0.5% dextrose, 0.0–0.4% urea and/or yeast extract w/v, pH 8.0] was made for each U+ candidate with the introduction of urea (1 M) as a nitrogen source over a concentration-dependent manner, substituting yeast extract. U+ candidates were grown in liquid B4 precipitation medium until reached log phase (∼1.0 A600 nm) and inoculated. Incubation conditions remained the same. After 12 weeks of incubation, the broth cultures were slowly poured off, laid flat, and placed on the viewing platform of a stereomicroscope (Kyowa Optical Sdz-Pl Zoom Microscope, Tokyo, Japan). Crystals were imaged at 40× using an iPhone 11 Pro (Apple Inc. Cupertino, United States).

### Petrographic and Scanning Electron Microscopy

Photomicrographs of closed 90-mL agar plates were taken using a Leica DMV6 digital microscope fitted with a Leica PlanApo FOV 12.55 mid-magnification objective that has a maximum field of view of 12.55 mm, magnification range from 46× to 675×, and working distance up to 33 mm (Leica, Germany).

Popcorn and soda straw speleothem samples were sent to the BioImaging Facility at the University of British Columbia, Vancouver, Canada for Scanning Electron Microscopy (SEM). Samples were freeze-dried and osmium-fumed in their storage tubes for approximately 1 h, and then transferred to polypropylene petri dishes with a saturated filter paper (4% w/v OSO_4_ aqueous solution) and 16% v/v formaldehyde (∼ 0.5 mL not touching the sample) for another 2 h. The samples were inverted after 1 h and further osmium-fumed over 48 h. Then, samples were mounted using a bulk sample holder and coated with 12 nm of gold using a Cressington 208 HR sputter coater (Cressington, Watford, United Kingdom). Samples were then imaged at various accelerating voltages, modes, and working distances on a Hitachi S4700 FESEM (Hitachi, Tokyo, Japan).

### Genetic Identification and Whole-Genome Sequencing

16s rRNA identification was performed by heating a single colony of each urease-positive isolate in 10 μL of nuclease-free water at 95°C for 10 min and using 2 μL of the resulting lysate for PCR amplification. An amplicon of approximately 570 bp was amplified using 1.25 U *Taq* DNA Polymerase (New England Biolabs), 0.5 mM dNTPs, 0.16 mM bovine serum albumin, 0.5 μM forward primer 5′-GTG CCA GCM GCN GCG G, and 0.5 μM reverse primer 5′-GGG TTN CGN TCG TTG. Cycling conditions were 95°C for 2 min, 25 cycles of 95°C for 1 min, 68°C for 45 s, and 68°C for 45 s, with final elongation at 68°C for 5 min. The resulting PCR product was Sanger-sequenced at Génome Québec (Montréal, Quebec, Canada). The sequencing results were trimmed, and genus-level identification was performed using NCBI Blast (Altschul et al., 1990), with one isolate identified to species level.

Two isolates with strong urease activity and observed carbonate-precipitation ability were selected for whole-genome sequencing (WGS). WGS was performed by Integrated Microbiome Technologies (Halifax, Nova Scotia, Canada) on the PacBio Sequel II instrument. Genome analyses were performed in the Galaxy platform (Afgan et al., 2018). Genomes were assembled using Flye Version 2.6 (Lin et al., 2016) and annotated using PATRIC Version 3.6.12 (Brettin et al., 2015). Quast Version 5.0.2 (Mikheenko et al., 2018) was used to analyze the quality of the assembled genome. PubMLST (accessed March 20, 2022) was then used to confirm genus-level identifications of the two isolates (Jolley et al., 2018).

### Chemical Analysis of Speleothems by Inductively Coupled Plasma Mass Spectrometry

Both PCS and SSS were ground into a slurry to determine their relative pH. Speleothem samples were ground using a sterile mortar and pestle into a fine powder (for SSS) and a wet slurry (for PCS). To the ground, SSS was added 6 mL of diH_2_O to form a slurry of similar consistency to PCS. Speleothem pH was determined using a FieldScout SoilStik pH meter (Spectrum Technologies, United States). SSS and PCS were also ground into fine powder and shipped to ALS Global Laboratories to perform inductively coupled plasma mass spectrometry (ICP-MS) total elemental analysis of sixteen elements, including biologically relevant elements such as magnesium and calcium.

## Results

### Isolation, Bioprecipitation, and Urease Activity

A total of 99 isolates were grown successfully in the lab setting. A total of four SSS isolates and seven PCS isolates exhibited urease activity of varying degrees while incubating at 9°C. The 11 total urease-positive isolates, henceforth known as U+ candidates, represent 8.2% of culturable sample size. Table 1 summarizes urease-positive candidates by showing how quickly the urea media changed color to intense pink, an indicator of increasing alkalinity. Isolates which had marginal urease activity were denoted as “+” (color change occurred 3–5 days post-inoculation), isolates with sufficient urease activity were denoted as “++” (color change occurred 2–3 days post-inoculation), and samples with generous urease activity were denoted as “+++” (color change occurred 0–1 days post-inoculation).

**TABLE 1:** Isolate designation, preliminary identification using 16S rRNA analysis, and urease activity.

Isolate Designation	Preliminary characterization	Activity
SSS006	*Sporosarcina* sp.	1++
SSS031	*Arthrobacter* sp.	+++
SSS032	*Arthrobacter* sp.	+++
SSS035	*Pseudarthrobacter* sp.	+++
PCS003	*Sphingobacterium anheuense*	++
PCS018	*Sphingobacterium* sp.	+
PCS039	*Streptomyces* sp.	++
PCS042	–	+++
PCS049	*Streptomyces* sp.	+++
PCS054	*Variovorax* sp.	++
PCS056	*Sphingobacterium* sp.	+++

*Isolates which had marginal urease activity were denoted as “+” (color change occurred 3–5 days post-inoculation), isolates with sufficient urease activity were denoted as “++” (color change occurred 2–3 days post-inoculation), and samples with generous urease activity were denoted as “+++” (color change occurred 0–1 days post-inoculation).*

**FIGURE 1:**
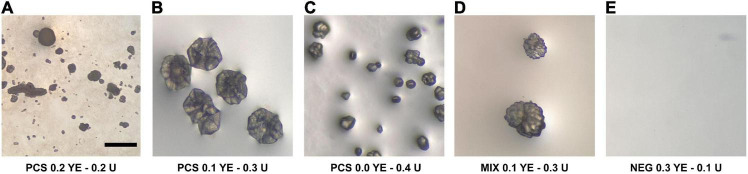
Crystal-like formations observed in modified liquid B4 media inoculated with isolates from the soda straw and popcorn speleothems observed by a stereomicroscope. **(A)** PCS056 (*Sphingobacterium* sp.) supplemented with 0.2% w/w yeast extract (YE) and 0.2% w/w urea (U). **(B)** PCS056 supplemented with 0.1% w/w YE and 0.3% w/w U. **(C)** PCS-56 supplemented with 0.0% w/w YE and 0.4% w/w U. **(D)** Mixed culture of SSS006 (*Sporosarcina* sp.) and SSS035 (*Pseudarthrobacter* sp.) supplemented with 0.1% w/w YE and 0.3% w/w U. **(E)** Negative control supplemented with 0.3% YE and 0.1% U. All micrographs under 40× magnification.

After 62 days from incubation, agar plates inoculated with U+ candidates demonstrated crystal formation on three of the four modified B4 media types. U+ candidates from both PCS and SSS grew on all media types; however, no growth was detected on B4 agar plates supplemented with calcium acetate for SSS U+ candidates only. Both PCS and SSS U+ candidates demonstrated crystal formations with varying morphologies. For the aqueous B4 experiment, broth cultures revealed crystal formations on the inside face of test tubes of three PCS and one SSS U+ candidates, respectively. For both the agar plate and aqueous B4 assays, U+ candidates, similar crystal morphologies, were seen, as demonstrated in Figure 1. SSS035 and PCS056 were chosen for whole-genome sequencing (WGS) based on their increased urease activity.

### Scanning Electron and Petrographic Microscopy

**FIGURE 2:**
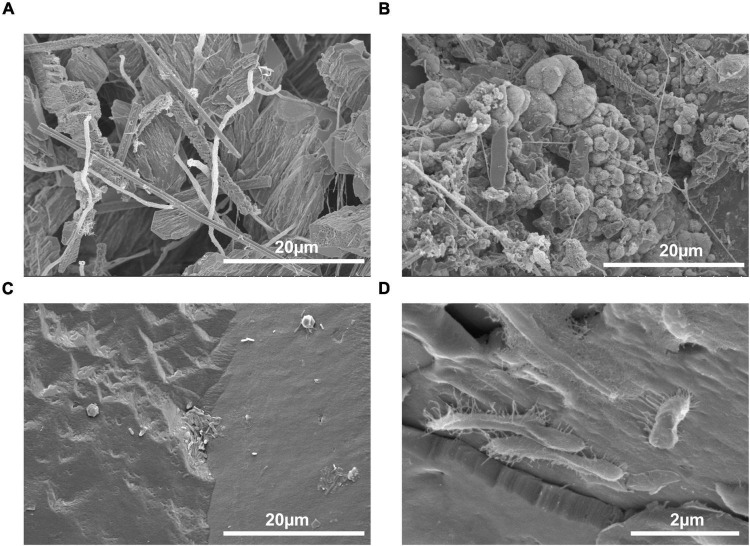
Scanning electron micrographs of speleothems with an abundance and variety of microorganisms present. **(A)** Inside of a popcorn speleothem sample (1000×), **(B)** outside of a popcorn speleothem sample (2500×), **(C)** internal base of a soda straw speleothem (2500×), and **(D)** exterior surface of a soda straw speleothem (1500×).

**FIGURE 3:**
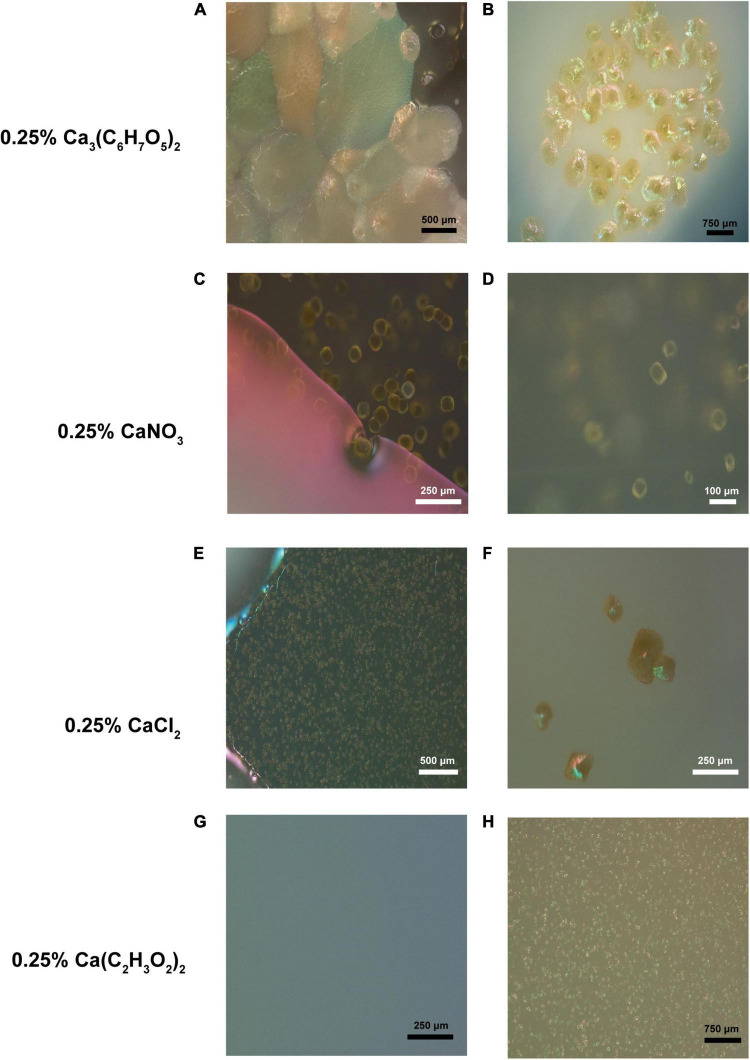
Crystal-like formations observed in various B4 media inoculated with isolates from the soda straw and popcorn speleothems observed by a petrographic microscope. **(A)** Formations observed adjacent a bacterial colony in the media of a B4 calcium citrate medium plate (131×). **(B)** Formations observed on the surface of a bacterial colony on calcium citrate B4 medium (69×). **(C)** Formations observed adjacent a bacterial colony in the media of a B4 calcium nitrate medium plate (343×). **(D)** Magnified formation on same colony as C (452×). **(E)** Crystals observed in calcium chloride B4 medium (151×). **(F)** Formations observed on calcium chloride B4 medium (306×). **(G)** Uninoculated B4 acetate media plate as a negative control (222×). **(H)** Crystals observed in calcium acetate B4 medium (91×).

A total of six micrographs were produced for both PCS and SSS by the Bioimaging Facility at the University of British Columbia (Figure 2). A total of eighty-two photomicrographs of SSS and PCS were taken with the petrographic microscope with an array of crystal structures, including rhombic and complex, as shown in Figure 3.

### Whole-Genome Sequencing and Phylogenetic Analysis

**FIGURE 4:**
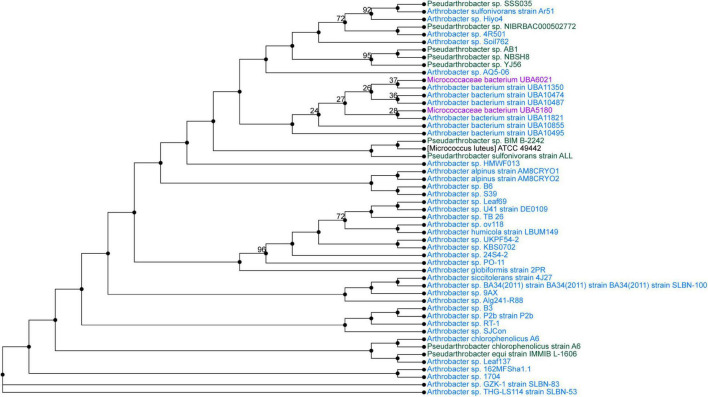
Phylogenetic tree from maximum likelihood analysis of bacterial whole-genome sequence. The numbers in branch points denote confidence levels of the relationship of sequences determined by bootstrap statistical analysis. *Pseudarthrobacter sp.* SSS035 is grouped with *A. sulfonivorans* strain Ar51 with maximum confidence level of 92.

*Pseudarthrobacter* sp. SSS035 and *Sphingobacterium* sp. PCS056 underwent WGS by PacBio Sequel II. The resulting reads were assembled using Flye and annotated using PATRIC. *Pseudarthrobacter* sp. SSS035 was assembled into three contigs with lengths of 4,611,850, 272,440, and 149,359 nucleotides. Average coverage of each contig was over 400×, with the average coverage of the three contigs being 537×. The total genome size was determined to be 5.03 Mbp and consisted of 64.86% GC content. PubMLST determined this isolate has an 82% match to *Pseudarthrobacter sulfonivorans*, indicating that this is likely a previously uncharacterized species of *Pseudarthrobacter.* Phylogenetic analysis of the most similar organisms by genome (Figure 4) associated the strain with *Arthrobacter* and *Pseudarthrobacter* species using NCBI Taxonomy tool (Schoch et al., 2020) was found in a diverse set of environments ranging from high humane activity areas such as coal mine and agricultural soil to wetlands on Tibetan Plateau lakes and tsunami-affected areas (Zhang et al., 2016; Park et al., 2019; Shen et al., 2021).

**FIGURE 5:**
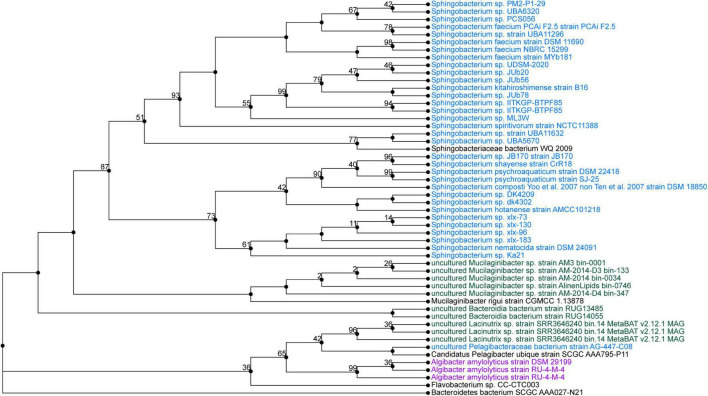
Phylogenetic tree from maximum likelihood analysis of bacterial whole-genome sequence. The numbers in branch points denote confidence levels of the relationship of sequences determined by bootstrap statistical analysis. *Sphingobacterium PCS056* was aligned with other representatives of the genus *Sphingobacterium*, with a confidence level of 67.

*Sphingobacterium* sp. PCS056 reads were assembled into a single contig with a length of 5,195,665 nucleotides with 36.78% GC content. Average coverage for this genome was 329×, and the genome was able to be circularized during assembly. PubMLST determined this isolate has an 80% match to *Sphingobacterium faecium*, also indicating the likelihood of a previously uncharacterized species. Phylogenetic analysis based on genome similarity (Figure 5) placed our isolate with other species of *Sphingobacterium* isolated from animal sources and agricultural soil such as pig pens and cow’s milk (Almeida et al., 2014; Ghosh et al., 2014), being very distinct from soil sources observed for the previous isolate described and cave bacteria in general.

**FIGURE 6:**
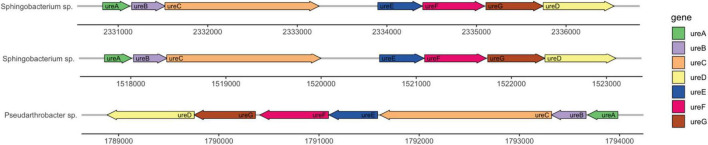
Urease gene clusters of cave bacterial isolates show similar genetic features. *Sphingobacterium* sp. PCS056 encodes two copies of the urease pathway genes (*ureABCDEFG*), with similar orientation and gene sizes, and a hypothetical gene in between *ureC* and *ureD*, represented as a gap. *Pseudarthrobacter* sp. SSS035 also presents a continuous cluster in a reverse orientation.

Genome annotation determined the presence of urease genes in each isolate (Figure 6). *Sphingobacterium* sp. PCS056 encodes the genes for alpha, beta, and gamma urease subunits, as well as the urease accessory proteins UreD, UreE, UreF, and UreG. Two gene clusters were observed, with similar genetic elements, but slightly different gene sizes. Upon further inspection, *Pseudarthrobacter* sp. SSS035 also has a full urease pathway encoding the same genes, running in the reverse sense. All gene clusters are 5 kb in size and are shown as a continuous pathway in *Pseudarthrobacter* sp. SSS035 as opposed to the ones observed in *Sphingobacterium* sp. PCS056, where an uncharacterized gene sequence is located in between *ureC* and *ureE*.

### Chemical Analysis of Speleothems

Both PCS and SSS were pH-analyzed in triplicate. PCS was found to be slightly acidic with a mean pH of 6.67, while SSS was found to be alkaline with a mean pH of 9.25.

A total of fifteen biologically relevant elements were analyzed by ICP-MS and conducted by ALS Global commercial laboratory. Table 2 summarizes the chemical profile of the samples. Of note, the largest elemental concentration among both SSS and PCS is calcium with concentrations of 411,000 mg/kg and 427,000 mg/kg, respectively. Further, nickel, which is responsible for binding urea and water in the active site of urease (Benini et al., 1999), was also present in both samples in concentrations of 1.88 mg/kg for SSS and 1.36 mg/mg for PCS.

**TABLE 2:** ICP-MS chemical analyses of PCS and SSS extracted from the ICC reported as concentration (mg/kg).

Element	Popcorn	Soda straw
Aluminum (Al)	604.00	76.00
Barium (Ba)	12.70	16.30
Cadmium (Cd)	1.12	0.43
Calcium (Ca)	427000.00	411000.00
Chromium (Cr)	1.77	< 0.50
Cobalt (Co)	< 0.10	<0.10
Copper (Cu)	3.63	0.56
Iron (Fe)	308.00	184.00
Lead (Pb)	< 0.50	<0.50
Magnesium (Mg)	309.00	1060.00
Molybdenum (Mo)	0.13	0.18
Nickel (Ni)	1.36	1.88
Potassium (K)	< 100	<100
Sodium (Na)	< 50	64.00
Strontium (Sr)	23.80	63.20
Zinc (Zn)	3.10	2.20

## Discussion and Conclusion

MICP is a general phenomenon demonstrated by bacteria among other microorganisms and is found in many habitats throughout the world. Though MICP as a process has been studied for over 20 years, research has focused on the ability for bacteria to precipitate calcium carbonate for use in biocements (Chou et al., 2011; Chu et al., 2012; Chaparro-Acuña et al., 2018; Seifan and Berenjian, 2019). However, research on bacterial-mediated MICP and how it plays an important role in shaping natural environments has focused mainly on terranean and subterranean soil studies. Research on MICP and how it contributes to cave formations is still not well known. Identifying whether MICP-capable bacteria play a role in the formation of cave speleothems and whether they contribute to the vastly different macroscopic morphologies exhibited by speleothems will help improve our understanding of bacterial-mediated MICP in subterranean environments. With vastly different speleothem morphologies, it is wondered whether bacteria act as nucleation sites for crystal growth, thus producing different crystal growth patterns. Further, with most speleothems being made of crystallized minerals, understanding how cave bacteria can survive and proliferate in such oligotrophic environments may well speak to their need to employ alternative metabolic pathways for energy, including those associated with MICP.

Here, we studied the MICP potential of cave bacteria isolated from the ICC in Chilliwack, Canada. Through a combination of biochemical, MICP-specific assays and genomics, we identified that MICP may be a common process found across different genera as demonstrated by bacteria isolated from PCS and SSS. MICP has been observed worldwide in a vast array of environments and microbial niches (Castanier et al., 1999; Stocks-Fischer et al., 1999; Chou et al., 2011; Seifan and Berenjian, 2019). Moreover, bacteria capable of MICP have participated in the formation of secondary cave deposits (Castanier et al., 1999; Stocks-Fischer et al., 1999; Northup and Lavoie, 2001; Engel et al., 2004; Banks et al., 2010). A previous study identified CaCO_3_ production in several genera of bacteria isolated from an Indian cave and further demonstrated their ability to precipitate on artificial B4 medium (Baskar et al., 2016). Furthermore, two genera isolated in the study by Baskar et al. (2016), *Arthrobacter* spp. and *Streptomyces* spp., were also identified in our study and were isolated from soda straw speleothems. Our results are consistent with MICP being a phenomenon demonstrated by bacteria found within caves and associated with speleothems (Figure 2) and is a process mediated by biochemical pathways found in a broad range of genera (Weiner and Dove, 2003; Zhu and Dittrich, 2016; Chaparro-Acuña et al., 2018; Castro-Alonso et al., 2019; Chaurasia et al., 2019; Seifan and Berenjian, 2019; Zhang et al., 2019; Ortega-Villamagua et al., 2020).

The bacterial isolates studied through WGS showed complete urease pathways, with some key differences in cluster structure and correlated species. Analysis of whole genome determined the closest correlated organisms to *Pseudarthrobacter* sp. SSS035 and *Sphingobacterium* sp. PCS056 (Figures 3, 4). *Pseudarthrobacter* sp. SSS035 was found to be related to other organisms commonly found in non-acidic soils and belonging to the same genus or the associated *Arthrobacter* genus with a high level of correlation confidence. Being known for their versatility in adapting to several conditions including desiccation, starvation, and the ability to use a wide range of carbon sources (Jones and Keddie, 2006), *Pseudarthrobacter* presence would be expected in a challenging cave environment. In our analysis, *Sphingobacterium* sp. PCS056 was related to other organisms from the same genus that are present in habitats with heavy human influence such as pig pens. Although the preferred habitat of the genus is not well characterized, the presence of several enzymes such as DNAse, oxidase, catalase, and urease, as well as antibiotic resistance genes (Steinberg and Burd, 2015), is indicative of adaptation to high evolutionary pressure conditions.

*Pseudarthrobacter* sp. SSS035 encodes a single and complete urease pathway, whereas *Sphingobacterium* sp. PCS056 shows two urease pathways encoded in the genome, with an unknown gene in the middle of the sequence (Figure 6). Gene duplications are common in prokaryotic organisms and can confer a variety of advantages. Duplications in genes related to transcription, defense mechanisms, and metabolism as we see in the urease pathway duplication can help the microbe adapt to a changing environment (Argyris and Pomerantz, 2004). Given that the cave environment is highly delicate and subject to small changes in nutrient availability, this could provide selective pressure to keep the duplicated pathway. Organisms that are better able to survive their environment due to gene duplication may experience convergent evolution toward the beneficial trait (Bratlie et al., 2010).

Fisher et al. (2017) found that in environmental bacteria, higher habitat pH correlated with higher copy numbers of *ureC.* The same trend occurs with the two isolates studied here, where duplication occurs in the isolate exposed to higher pH. However, we do see duplication of the entire pathway rather than just in *ureC*, in a similar manner to several *Helicobacter* species, where the urease is associated with host iron-rich diets and presents reduced activity (Carter et al., 2009, 2011; Fong et al., 2013). Considering the cave high levels of iron, hence its name, this duplication, and the altered configuration with a hypothetical gene between *ureC* and *ureD* could arise from the presence of an alternative iron-dependent urease(s) in addition or in replacement of nickel-dependent enzymes due to limited nickel levels in comparison with iron (Table 2).

The urease pathway occurring in the reverse direction in *Pseudarthrobacter* sp. SSS035 could be due to a variety of reasons, including differences in operon regulation or convergent evolution with the urease genes arising in the two microbes based on two separate events. One study proposes that inversion of whole operons occurs more often in genes associated with antibiotic resistance and virulence and increases evolvability of the microbe to better adapt to its environment and lifestyle (Merrikh and Merrikh, 2018). The pathogenicity of *Pseudarthrobacter sulfonivorans*, the closest related bacteria to *Pseudarthrobacter* sp. SSS035, is undetermined, but there are members of the genus that are known to be pathogenic, supporting the possibility of another pathogenic microbe in this genus.

In addition, we studied the chemical composition of the PCS and SSS speleothems to determine differences in available nutrients and in the immediate microenvironment that bacteria inhabited. Recent studies by Dhami et al. (2018) have analyzed the chemical composition of groundwater and analyzed six speleothems from Margaret River caves to elucidate what minerals and free aqueous species are available to cave-dwelling bacteria. Interestingly, a similar trend was observed between the three caves sampled in the study and our observations at the ICC with respect to calcium, iron, and magnesium, despite the geographic distance between Margaret Rivers in Australia and ICC in Canada. Likewise, similar trends in trace elements demonstrate that a similar chemical environment would be available to cave-dwelling bacteria.

Dhami et al. (2018) also performed metagenomic sequencing and reported an amplicon library where all five phyla and two genera specific (*Pseudarthrobacter* sp. and *Sporosarcina* sp.) U+ candidates from the ICC were found in the Australian caves, further cementing the idea that the microenvironment (i.e., available dissolved minerals and drip water on speleothem surfaces) may be an important selective pressure, favoring these genera. To corroborate these findings, the results from a study by Ortiz et al. (2012) on the bacterial diversity in the Kartchner Cave in Arizona, United States, demonstrated that bacteria belonging to all five phyla found at the ICC were also present in the Kartchner Cave. These studies suggest that these organisms might utilize alternative metabolic pathways to inhabit and persist in such oligotrophic environments under different environmental variables all over the world, consolidating the importance of urease in our isolates for cave survival.

While bacterial abundance may allow us to understand the community composition, we must not forget the role the pH plays in both the geological and biological worlds. We have a good understanding of how caves form, and the role that pH plays; however, we do not have a great understanding of the role pH plays in the microenvironment that cave bacteria inhabit. In this study, PCS was determined to be slightly acidic pH, while SSS was alkaline. With these findings, PCS likely undergo passive influx of calcium ions and SSS undergo active extrusion of calcium ions for calcium carbonate precipitation as demonstrated in a study conducted by Hammes and Verstraete (2002) whereby researchers outlined mechanisms by which bacteria participate in active and passive calcium carbonate precipitation thereby demonstrating a clear mechanistic approach.

Lastly, it is imperative to understand the role that solid mineral surfaces of speleothems and their aqueous environments play in the physical geological formation of crystals. Calcium carbonate crystal formations exist as either calcite, aragonite, or vaterite. Here, we employed petrographic microscopy to discern what crystal shapes were formed from MICP of U+ candidates, and whether the crystal shapes differed between the solid and liquid MICP assays. A wide array of crystal morphologies was found upon imaging U+ candidates from both liquid and solid MICP assays, as demonstrated in Figures 1, 3. Regarding crystal morphology, even though we understand that the conditions between *in vitro* experiments vs. cave environments differ greatly, discrepancies in the observed shapes of crystal made by different single isolates vs. mixed cultures were observed. Hence, we are convinced that the makeup of bacteria may influence the diversity in crystal morphology and may be related to the macroscopic shape of the speleothems themselves, suggesting that crystal budding from urease-positive candidates may influence a larger exponentiation of the crystal growth, aided along by either evaporation pattern and/or dripping of the groundwater saturated with mineral contents, to form the macroscopic speleothems that we see today.

Since speleothem formation is very complex and time-consuming process, lots of metabolic pathways and steps involved in cave speleothem formation remain unknown. From our lab-based crystal formations found in U+ candidate MICP assays, we now have many more questions about it than we started off. For example, we wonder whether the growth of speleothems and their structures are first nucleated at the extracellular surface of cave bacteria that precipitate calcium carbonate. How about the pH role and impact in an overall crystal formation relating to the bacterial cell membrane and subsequent cascading of proton gradients, and its effects in the microenvironment? These questions warrant us to look further into how bacterial diversity and activity influence or are influenced by the cave speleothem dynamics.

Prospects include determining the nucleation role of cave bacteria in speleothem growth by performing carbonatogenic yield experiments to better understand growth kinetics of U+ candidates, and their quantitative contribution to overall speleothem formation. Moreover, determining the quantity of nucleation sites on these candidates is imperative but requires careful assessment of the number of urease enzyme copies found in their genomes and the available surface area to act as nucleation sites. Determining surface area nucleation sites requires further imaging with SEM when bacteria are in their ureolytic metabolic state. Lastly, to confirm the crystal structure being produced by U+ candidates is indeed the calcite form of calcium carbonate, X-ray diffraction microscopy must be employed.

## Data Availability Statement

The datasets presented in this study can be found in online repositories. The names of the repository/repositories and accession number(s) can be found in the article/Supplementary Material.

## Author Contributions

NC, SL, and RM obtained funding. NC, KK, and RM conceived the study and designed the experiment. KK, RM, LC, and SL conducted the experiments. KK, RM, JG, and NC designed the MS workflow. KK, RM, SL, and JG were instrumental in collecting the MS data sets. KK, RM, and JG drafted the manuscript. NV and DH assisted with microscopy. NC, NV, KK, RM, SL, and JG read and edited the manuscript. CB was JG’s Ph.D. supervisor. All authors contributed to the article and approved the submitted version.

## Conflict of Interest

The authors declare that the research was conducted in the absence of any commercial or financial relationships that could be construed as a potential conflict of interest.

## Publisher’s Note

All claims expressed in this article are solely those of the authors and do not necessarily represent those of their affiliated organizations, or those of the publisher, the editors and the reviewers. Any product that may be evaluated in this article, or claim that may be made by its manufacturer, is not guaranteed or endorsed by the publisher.

## Supplementary Material

Supplementary Figure 1 Map of the Iron Curtain Cave demonstrating the location of the two speleothem collection sites. **(A)** PCS sampling and **(B)** SSS sampling. Locations are approximate.

Supplementary Figure 2 Examples of speleothems present in ICC collected for this study. **(A)** Popcorn speleothem. **(B)** Soda straw speleothem.
