# Supplementary figures and images for: Biomineralization in Cave Bacteria—Popcorn and Soda Straw Crystal Formations, Morphologies, and Potential Metabolic Pathways
Source: Front Microbiol. 2022 Jul 1;13:933388. doi: 10.3389/fmicb.2022.933388 (PMC9283089; doi:10.3389/fmicb.2022.933388)

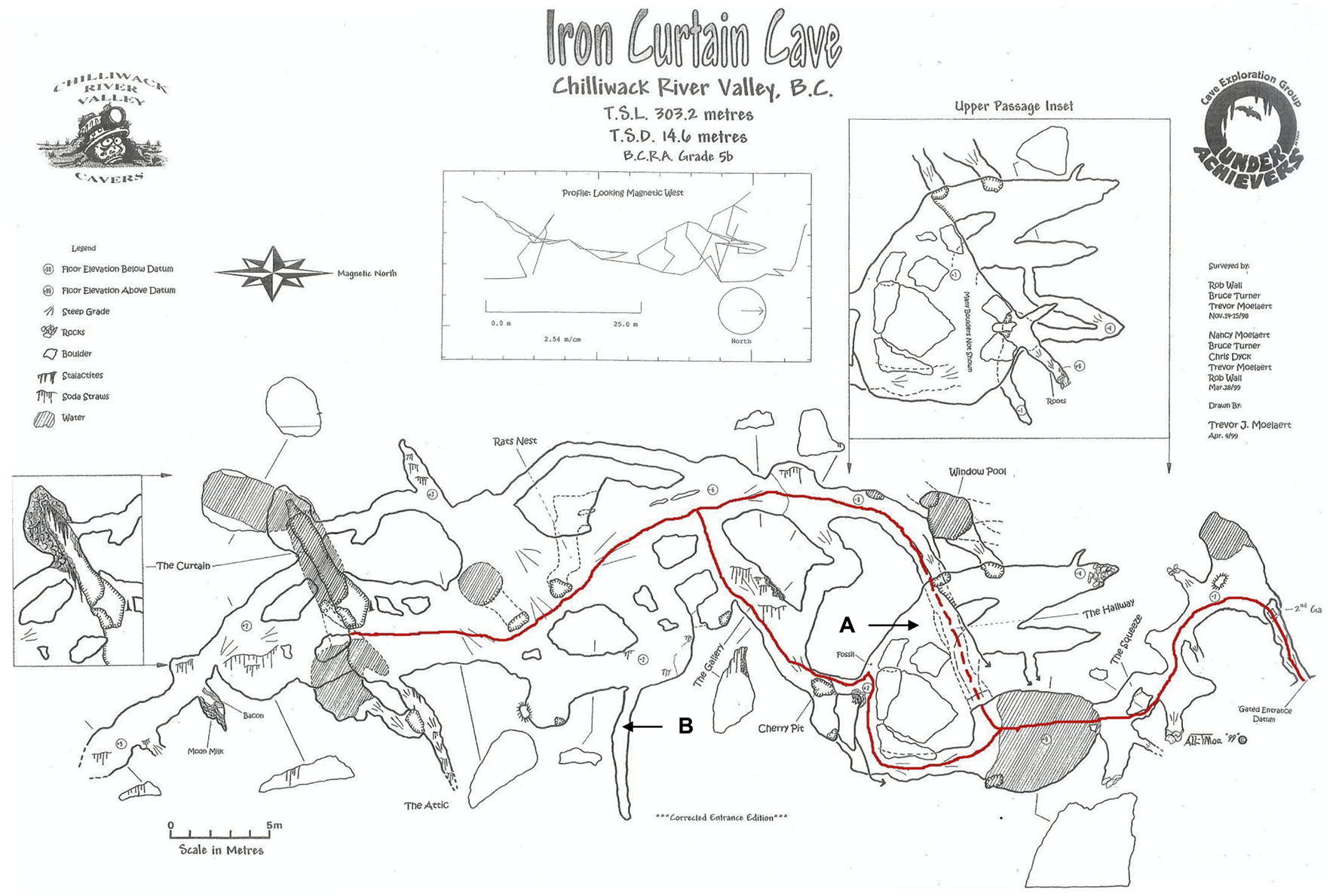

Supplement: Supplementary Figure 1 — Map of the Iron Curtain Cave demonstrating the location of the two speleothem collection sites. (A) PCS sampling and (B) SSS sampling. Locations are approximate. [file Image_1.TIF]

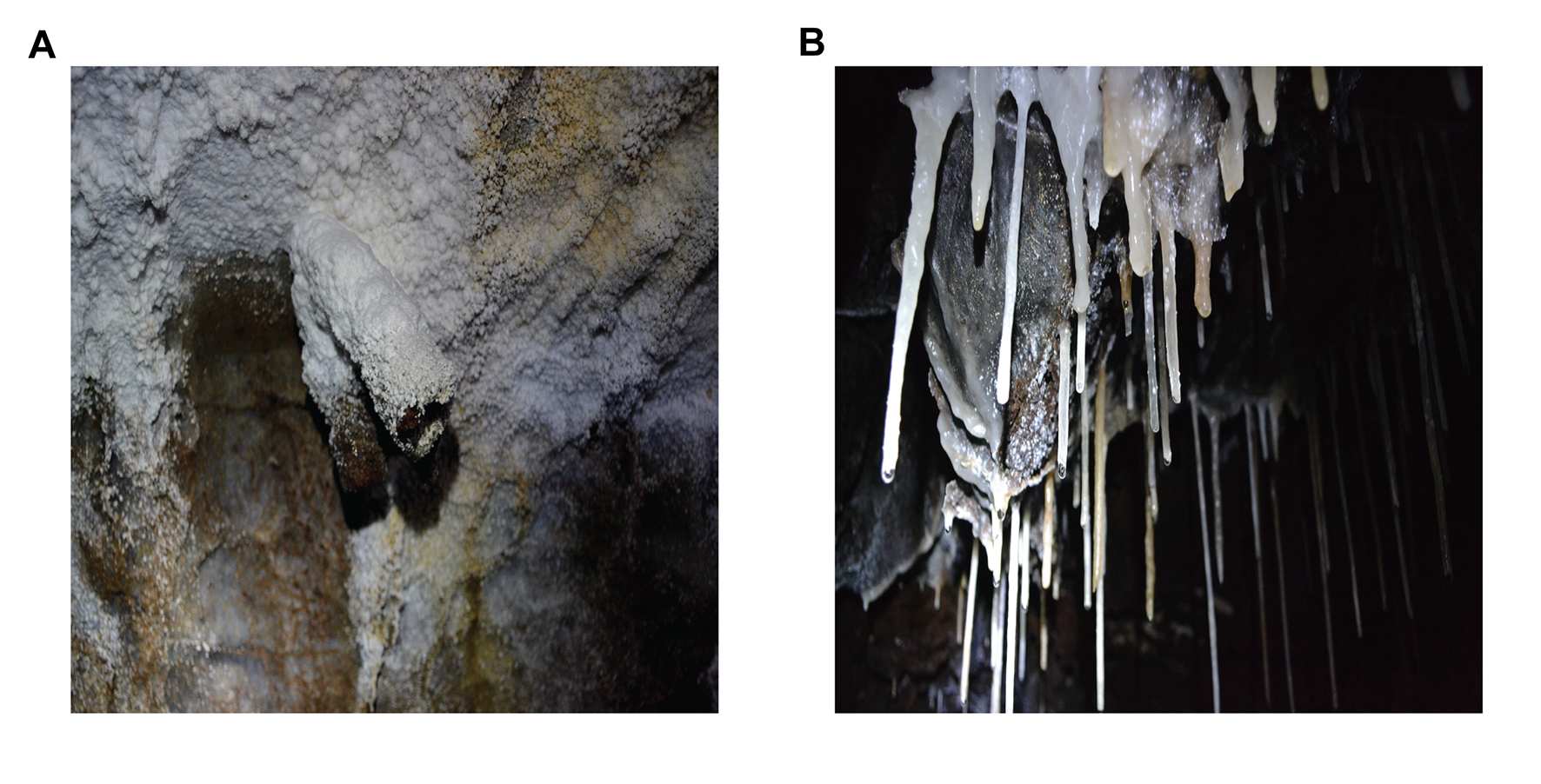

Supplement: Supplementary Figure 2 — Examples of speleothems present in ICC collected for this study. (A) Popcorn speleothem. (B) Soda straw speleothem. [file Image_2.TIF]
